# Research hotspots and frontiers of preconditioning in cerebral ischemia: A bibliometric analysis

**DOI:** 10.1016/j.heliyon.2024.e24757

**Published:** 2024-01-21

**Authors:** Long Zhang, Xue Zhou, Jing Zhao, Xingchen Wang

**Affiliations:** a First Clinical Medical College, Shandong University of Traditional Chinese Medicine, Jinan 250355, China; b Experimental Center, Shandong University of Traditional Chinese Medicine, Jinan 250355, China; c Division of Neurology, The Second Affiliated Hospital of Shandong University of Traditional Chinese Medicine, Jinan, 250001, China; d Department of Traditional Chinese Medicine, Zibo TCM-Integrated Hospital, Zibo ,255026, China

**Keywords:** Preconditioning, Ischemic tolerance, Cerebral ischemia, Mechanisms, Remote ischemic preconditioning, Preconditioning of mesenchymal stem cells, CiteSpace

## Abstract

**Background:**

Preconditioning is a promising strategy against ischemic brain injury, and numerous studies in vitro and in vivo have demonstrated its neuroprotective effects. However, at present there is no bibliometric analysis of preconditioning in cerebral ischemia. Therefore, a comprehensive overview of the current status, hot spots, and emerging trends in this research field is necessary.

**Materials and methods:**

Studies on preconditioning in cerebral ischemia from January 1999–December 2022 were retrieved from the Web of Science Core Collection (WOSCC) database. CiteSpace was used for data mining and visual analysis.

**Results:**

A total of 1738 papers on preconditioning in cerebral ischemia were included in the study. The annual publications showed an upwards and then downwards trend but currently remain high in terms of annual publications. The US was the leading country, followed by China, the most active country in recent years. Capital Medical University published the largest number of articles. Perez-Pinzon, Miguel A contributed the most publications, while KITAGAWA K was the most cited author. The focus of the study covered three areas: (1) relevant diseases and experimental models, (2) types of preconditioning and stimuli, and (3) mechanisms of ischemic tolerance. Remote ischemic preconditioning, preconditioning of mesenchymal stem cells (MSCs), and inflammation are the frontiers of research in this field.

**Conclusion:**

Our study provides a visual and scientific overview of research on preconditioning in cerebral ischemia, providing valuable information and new directions for researchers**.**

## 1 Introduction

Cerebral Ischemia is the reduction or blockage of blood flow to brain tissue due to arterial occlusion or systemic hypoperfusion, usually accompanied by hypoxia. Severe cerebral ischemia leads to lethal injury and ultimately disability or death, greatly endangering human health. Ischemic stroke is the most common form of cerebral ischemia, accounting for 87 % of strokes [1]. Stroke is the second leading cause of death worldwide [2]. On average, a stroke occurs every 40 s in the United States [1]. In 2020, there were 17.8 million cases of stroke in China among people over 40 years old, including 3.4 million new strokes. There were 2.3 million stroke-related deaths that year, presenting an enormous challenge [3]. Pharmacological thrombolysis and mechanical thrombolysis have been clinically successful but have limited applications. Therefore, researchers have been exploring new strategies to better mitigate ischemic injury.

Interestingly, it was observed that brief mild ischemia not only did not cause lethal damage and also improved the body's resistance to subsequent severe ischemia [4,5]. This sublethal ischemic insult is known as ischemic preconditioning (IPC), and the protection against lethal ischemic insults is known as ischemic tolerance (IR) [6]. In 1990, KITAGAWA from Japan first reported on the phenomenon of ischemic tolerance in the brain [7], giving impetus to the development of the field. As an endogenous protection strategy, preconditioning has received worldwide attention. Numerous in vitro and in vivo studies have confirmed the effective neuroprotection of preconditioning [8,9]. The scope of preconditioning has also expanded from the initial ischemia to other biological, physical, and chemical stimuli [10]. Therefore, it is essential to review and analyse the published articles on preconditioning in cerebral ischemia to help researchers identify the developmental lines and research hotspots.

As an effective tool for analysing large amounts of academic literature, bibliometrics can be used qualitatively and quantitatively to uncover valid information in the literature from different dimensions [11]. Bibliometrics can help researchers to objectively assess the contributions of countries, institutions, authors, and journals in a given field and to quickly understand the knowledge structure, focus, and emerging trends. CiteSpace is a visual bibliometric software invented by Professor Chaomei Chen that has been widely used in medical research [[12], [13], [14]]. CiteSpace consists of three modules: the Collaborative Network, the Co-occurrence Network, and the Cocitation Network. Collaboration networks and author cocitation networks allow the visual analysis of the contribution and collaboration of countries, institutions, and individuals in the field. The keyword co-occurrence network and the cocited references network constitute the knowledge base, which can be visually analyzed to identify the current research status and hot spots. Burst detection and clustering timeline maps can help researchers understand the evolution of research hotspots and emerging research trends. There is no bibliometric analysis of preconditioning in cerebral ischemia.

In this study, we conducted the first bibliometric analysis of the literature collected from WOS using CiteSpace software to provide a comprehensive overview of research advances in preconditioning in cerebral ischemia. This study was conducted with three objectives. First, we aimed to visualize and analyse the contributions and collaborations of countries, institutions, and individuals. Second, we summarized the knowledge base and research hotspots. Third, we explored new hotspots and research trends.

## 2 Materials and methods

### 2.1 Data acquisition

**Fig. 1:**
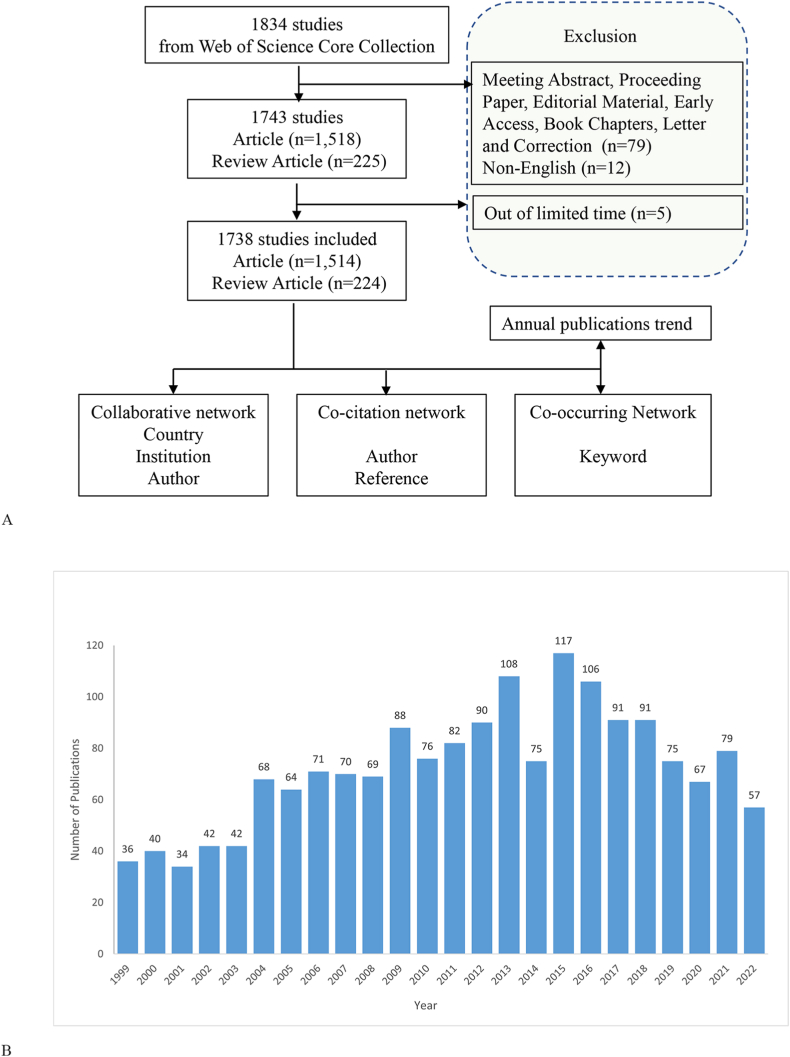
(A)Research flow chart (B)Annual publications trends chart.

All relevant studies were collected from the Web of Science Core Collection (WOSCC) on May 5th, 2023. The following search strategy was selected: #1 TS=("Cerebral Ischemi*" OR "Brain Ischemia*" OR "Ischemic Encephalopath*" OR "Cerebral Infarction*" OR "Cerebral Infarct*" OR "Subcortical Infarction*" OR "Posterior Choroidal Artery Infarction*" OR "Anterior Choroidal Artery Infarction*" OR "Ischemi* Stroke*" OR "Ischaemic Stroke*" OR "Cryptogenic Ischemic Stroke*" OR "Cryptogenic Stroke*" OR "Cryptogenic Embolism Stroke*"OR "Acute Ischemic Stroke*"), #2 TS=("Preconditioning*" OR "Pre-Conditioning*" OR "Pre Conditioning*"), #1 & #2. Timespan: From January 1st, 1999, to December 31st, 2022. Document type: article or review article. Language: English. Finally, a total of 1743 publications were obtained (Fig. 1A). All article records were exported in the plain text file format of “Full Records and Cited References”.

### 2.2 Analysis tool

CiteSpace 6.2.R2 (Advanced) was used to perform a bibliometric analysis. A total of 1,738 article records were left after removing duplicates and articles published in 2023. The following parameters were selected: time slicing (from January 1999 to December 2022), years per slice (one), term source (full selection), node type (choose as needed), links (default), selection criteria (g-index, k = 25), and pruning (Pathfinder, pruning the merged network).

## 3 Result

### 3.1 Trends of annual publications

A total of 1,738 papers published from 1999 to 2022 were analyzed for annual volume. As shown in Fig. 1B, the number of publications in this field was less than 50 per year, with a very slow growth rate from 1999 to 2003. There has been a significant increase since 2004, particularly from 2012 to 2018, when the number of annual publications exceeded 90 (except in 2014); this suggests that the subject attracted more attention from scholars during this period. The number of publications reached a peak of 117 in 2015. Although there has been a slight downwards trend since 2019, the number of articles was still above 50 per year, indicating that scholars have maintained an interest in in-depth research.

### 3.2 Visual distribution of countries (regions) and institutions

**Fig. 2:**
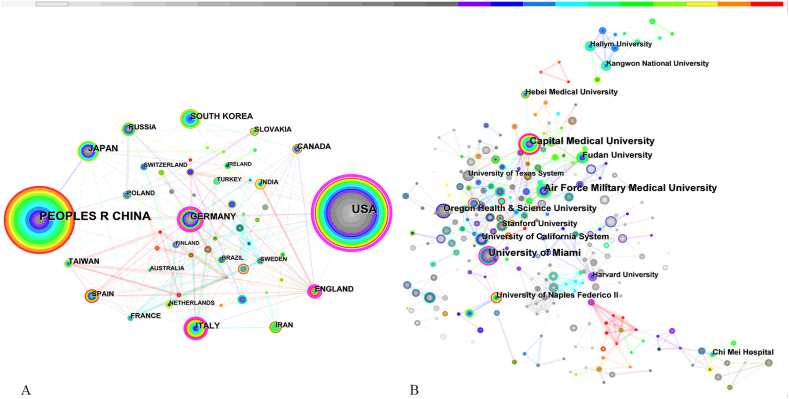
(A)The collaborative network of countries (regions). (B) The collaborative network of institutions.

The collaborative network of countries (regions) was generated by CiteSpace (Fig. 2A). The network map had a total of 53 nodes, 201 links, and a density of 0.1459. Nodes represent countries (regions). There was a positive correlation between node size and count, the larger the node size was, the higher the count. Both the United States (581 articles) and China (546 articles) contributed more than 500 articles, accounting for over 50 % of the total, followed by Japan, Germany, Italy, South Korea, England, Taiwan, Canada, and Slovakia (Table 1). The nodes coloured purple round have higher betweenness centrality values (≥0.10). The top 4 countries in terms of betweenness centrality were the United States (0.61), Germany (0.30), England (0.21), and Italy (0.18).

**Table 1:** Top 10 high-productivity countries (regions) and top 4 countries (regions) with the highest centrality.

Rank	Country /Region	Publications	Centrality	Rank	Country /Region	Centrality	Publications
1	USA	581	0.20	1	USA	0.61	581
2	PEOPLES R CHINA	546	0.07	2	GERMANY	0.30	98
3	JAPAN	107	0.14	3	ENGLAND	0.21	49
4	GERMANY	98	0.23	4	ITALY	0.18	73
5	ITALY	73	0.27				
6	SOUTH KOREA	66	0.00				
7	ENGLAND	49	0.48				
8	TAIWAN	48	0.00				
9	CANADA	47	0.07				
10	SLOVAKIA	39	0.14				

The distribution network of institutions involved in this research is shown in Fig. 2 (B). Node = 453, Link = 748, Density = 0.0073. Each node represents an institution. The top 10 institutions are listed in Table 2. Capital Medical University from China ranked first with 64 articles published, followed by University of Miami (58 articles), Air Force Military Medical University (55 articles), Oregon Health & Science University (36 articles), University of California System (32 articles), Fudan University (30 articles), University of Naples Federico II (26 articles), Stanford University (25 articles), Chi Mei Hospital (23 articles) and Hebei Medical University (21 articles). Despite being in 11th place in terms of numbers, Harvard University was at the top in terms of betweenness centrality (0.29), indicating that it had a very strong influence in this field and collaborated widely with other institutions. The remaining institutions with high centrality (>0.1) were Capital Medical University (0.17), the University of Miami (0.16), and the University of Lyon (0.15).

**Table 2:** Top 10 high-productivity institutions and top 4 institutions with the highest centrality.

Rank	Institution	Publications	Centrality	Rank	Institution	Centrality	Publications
1	Capital Medical University	64	0.17	1	Harvard University	0.29	20
2	University of Miami	58	0.16	2	Capital Medical University	0.17	64
3	Air Force Military Medical University	55	0.03	3	University of Miami	0.16	58
4	Oregon Health & Science University	36	0.01	4	CHU Lyon	0.15	3
5	University of California System	32	0.09				
6	Fudan University	30	0.04				
7	University of Naples Federico II	26	0.01				
8	Stanford University	25	0.01				
9	Chi Mei Hospital	23	0.01				
10	Hebei Medical University	21	0				

### 3.3 Analysis of author collaboration network

**Fig. 3:**
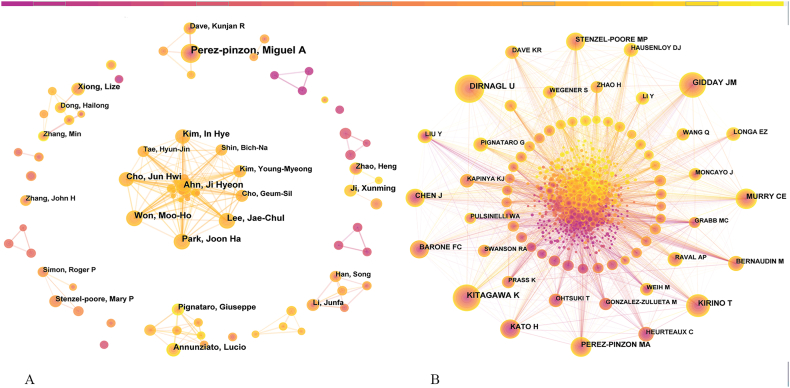
(A) The collaborative network of authors. (B) The network of cocited authors.

A coauthor map with 809 nodes and 1,357 links was generated by CiteSpace (Fig. 3A). In this collaboration network, different nodes represented different authors and the line between nodes represented the collaboration between two authors. Some scientific research teams are clearly represented in Fig. 3 (A). Table 3 shows the top 11 authors, including Perez-Pinzon, Miguel A (38 articles), Ahn, Ji Hyeon (21 articles), Lee, Jae-Chul (19 articles), Won, Moo-Ho (19 articles), Park, Joon Ha (19 articles), Kim, In Hye (17 articles), Cho, Jun Hwi (17 articles), Annunziato, Lucio (16 articles), Ji, Xunming (14 articles), Pignataro, Giuseppe (14 articles) and Xiong, Lize (14 articles). The year referred to the year in which the author first published the paper in Table 3. During the timespan we chose, Perez-Pinzon, Miguel A from the University of Miami showed great enthusiasm for this scientific research from the beginning. His research group focused on the molecular mechanisms of neuroprotection resulting from ischemic preconditioning. Since 2014, a new scientific research team from South Korea, mainly including Ahn Ji Hyeon, Won Moo-Ho, Lee Jae-Chul, Park Joon Ha, Kim In Hye, and Cho Jun Hwi, has published a number of articles on this research topic, forming an enormous huge cooperative network. These authors mainly used the gerbil model to study the mechanism of neuroprotection of ischemic preconditioning.

**Table 3:** Top 11 high-productivity authors and top 10 cocited authors.

Rank	High-Productive Author	Publications	Year	Rank	Co-Cited Author	Citations	Year
1	Perez-pinzon, Miguel A	38	1999	1	KITAGAWA K	452	1999
2	Ahn, Ji Hyeon	21	2014	2	DIRNAGL U	425	2001
3	Lee, Jae-Chul	19	2014	3	GIDDAY JM	367	1999
3	Won, Moo-Ho	19	2014	4	KIRINO T	339	1999
3	Park, Joon Ha	19	2014	5	MURRY CE	260	1999
6	Kim, In Hye	17	2014	6	PEREZ-PINZON MA	247	1999
6	Cho, Jun Hwi	17	2014	7	CHEN J	227	1999
8	Annunziato, Lucio	16	2009	8	KATO H	216	1999
9	Ji, Xunming	14	2012	9	BARONE FC	192	1999
9	Pignataro, Giuseppe	14	2013	10	STENZEL-POORE MP	189	2004
9	Xiong, Lize	14	2009				

### 3.4 Analysis of co-cited authors

Another way to study the impact of authors on the field is through the analysis of cocited authors. We used CiteSpace to generate the author cocitation map. As presented in Fig. 3 (B), there were 979 nodes and 7,889 links. The largest node in this map was KITAGAWA K from Japan, which was cited 452 times. In 1991, he and his group demonstrated that ischemic preconditioning could prevent neuronal death after global ischemia, and first proposed the concept of ischemic tolerance in the brain, launching the field of cerebral ischemic tolerance research. The top 10 co-cited authors are listed in Table 3, among which there were 8 scholars cocited over 200 times, including KITAGAWA K (452 citations), DIRNAGL U (425 citations), GIDDAY JM (367 citations), KIRINO T (339 citations), MURRY CE (260 citations), PEREZ-PINZON MA (247 citations), CHEN J (227 citations) and KATO H (216 citations).

### 3.5 Analysis of co-cited references

**Fig. 4:**
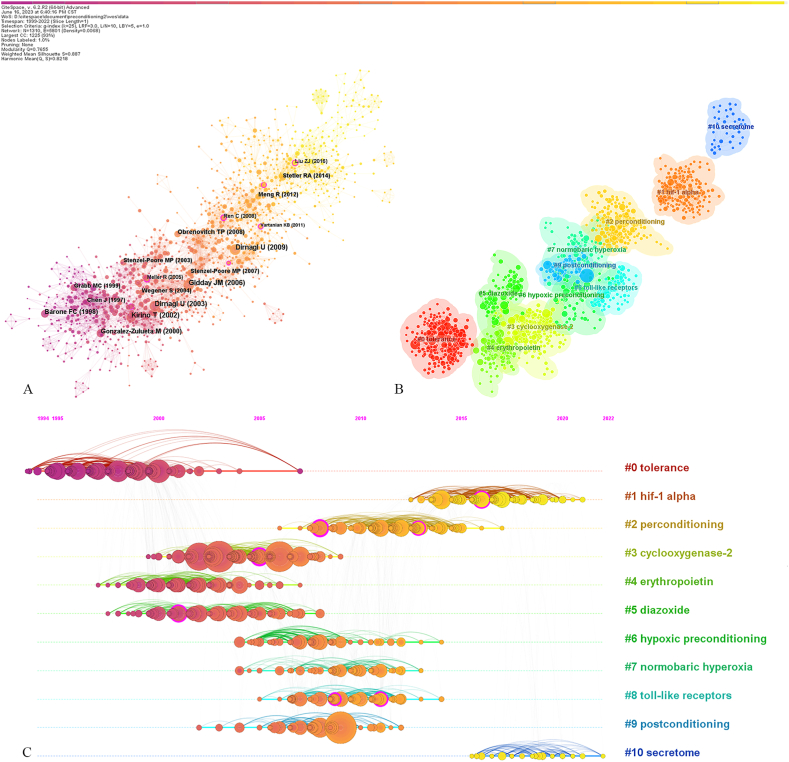
(A) The network of cocited references. (B) The cluster map of cocited references. (C) The timeline map of cocited references.

Generating a cocited reference network map (Fig. 4A) resulted in 1,310 nodes and 5,801 links. Each node corresponded to one cocited reference and the larger the node, the more cocitations there were. Table 4 lists the top 10 cocited references, consisting of 6 reviews and 4 articles. The first and third high co-citated references were both reviews published by Dirnagl U from Germany. The review (2003) published in TRENDS NEUROSCI (IF:15.9) provided an overview of the understanding of cerebral ischemic preconditioning (or ischemic tolerance), summarized the triggers, temporal profiles, and endogenous neuroprotective mechanisms, and then pointed out a few open issues and key challenges [15]. Both substrate restriction and noxious events are capable of inducing IPC/IT. Substrate delivery, energy metabolism, anti-excitotoxicity, anti-inflammation, and anti-apoptosis are involved in endogenous neuroprotection. A further review (2009) published in LANCET NEUROL (IF:48.0) added different types of ischemic preconditioning, introduced genomic reprogramming conferring cytoprotection and survival, summarized the effects of the key transcription factor HIF (hypoxia-inducible factor), elaborated four basic actions capable of improving outcome after ischemic events, and categorized the molecular signalling cascades of induced neuroprotection according to sensor, transducers, and effectors [9]. In addition, the authors summarized the advances, potential problems, and challenges of ischemic preconditioning in clinical practice. The fifth high-frequency co-cited reference was an original research article published by Barone FC in STROKE (IF:8.3). In this study, local ischemic preconditioning (single middle cerebral artery occlusion (MCAO) for 10 min) significantly reduced the infarct size of the ipsilateral hemisphere if it was performed 1–7 days before permanent MCAO [16]. This neuroprotection was associated with protein synthesis, upregulation of interleukin-1 receptor (IL-1R) antagonist, and downregulation of the early response genes c-fos and zif268. Single ischemic preconditioning did not decrease contralateral cortical injury. In another study with high-frequency cocitations published by Gonzalez-Zulueta M (IF:11.1), oxygen-glucose deprivation (OGD) preconditioning (5 min) in vitro exhibited powerful neuroprotective effects [17]. It was demonstrated that the p21^ras^ (Ras)/extracellular regulated kinase (Erk) pathway initiated by activation of N-methyl-d-aspartic acid (NMDA) receptors and production of nitric oxide (NO) played a key role in the development of OGD tolerance. A study with microarray analysis published in LANCET (IF:168.9) revealed that complex genomic reprogramming served as a significant regulatory mechanism of neuroprotection induced by ischemic/OGD preconditioning [18]. Preconditioning elicited transcriptional changes involved in metabolic suppression, immunosuppression, inhibition of ion-channel activity, and hypocoagulation. These neuroprotective strategies were similar to those in hibernation and hypoxia-tolerant states.

**Table 4:** Top 10 cocited references with the highest frequency.

Rank	Co-cited reference	Frequency	Centrality	Impact Factor	Type
1	Dirnagl U, 2009, LANCET NEUROL,https://doi.org/10.1016/S1474-4422(09)70054-7	90	0.01	48.0	Review
2	Gidday JM, 2006, NAT REV NEUROSCI, https://doi.org/10.1038/nrn1927	87	0.02	34.7	Review
3	Dirnagl U, 2003, TRENDS NEUROSCI, https://doi.org/10.1016/S0166-2236(03)00071-7	83	0.01	15.9	Review
4	Kirino T, 2002, J CEREBR BLOOD F MET, https://doi.org/10.1097/01.WCB.0000040942.89393.88	71	0.02	6.3	Review
5	Barone FC, 1998, STROKE, https://doi.org/10.1161/01.STR.29.9.1937	46	0.05	8.3	Article
6	Gonzalez-Zulueta M, 2000, P NATL ACAD SCI USA, https://doi.org/10.1073/pnas.97.1.436	46	0.02	11.1	Article
7	Obrenovitch TP, 2008, PHYSIOL REV, https://doi.org/10.1152/physrev.00039.2006	39	0.03	33.6	Review
8	Stenzel-Poore MP, 2003, LANCET, https://doi.org/10.1016/S0140-6736(03)14412-1	37	0.01	168.9	Article
9	Stenzel-Poore MP, 2007, STROKE, https://doi.org/10.1161/01.STR.0000251444.56487.4c	35	0.06	8.3	Review
10	Grabb MC, 1999, J NEUROSCI, https://doi.org/10.1523/JNEUROSCI.19-05-01657.1999.	35	0.04	5.3	Article

Node with a purple ring has a high betweenness centrality value (≥0.10), which is usually regarded as a significant tipping point connecting different point groups in the network. There were 7 references (Table 5) serving as tipping points, most of which are original research papers. The earliest article (centrality value 0.10) published in 2001 demonstrated that upregulation of B-cell lymphoma-2(Bcl-2) expression was a key mechanism of ischemic tolerance induced by local ischemic preconditioning [19]. Later in 2005, another study (centrality value 0.34) further revealed that the activation of cAMP response element binding protein (CREB) upregulated Bcl-2 protein expression as an essential step of ischemic preconditioning to protect against ischemic injury [20]. These two articles both focused on the Bcl-2-mediated neuroprotection against apoptosis, using a local ischemic preconditioning model in vivo. The research paper with the highest centrality value (0.39) published in 2008 confirmed that repeated remote ischemic preconditioning (RIPC) significantly reduced infarct size in a focal ischemia model in rats [21]. Researchers found that the protection induced by RIPC depended on the cycle numbers of occlusion/reperfusion and time windows. Moreover, the therapeutic time windows were different between RIPC and conventional preconditioning. In a review (centrality value 0.13) published in 2009, the authors investigated the key role of Toll-like receptors (TLRs) in cerebral ischemic injury and indicated that the genomic reprogramming of TLR signaling induced by preconditioning conferred strong neuroprotection, including downregulation of proinflammatory mediators and upregulation anti-inflammatory mediators [22]. The study (centrality value 0.10) published in 2011 focused on the inflammatory mechanisms of neuroprotection induced by lipopolysaccharide (LPS) preconditioning in the TLR4/TRIF signalling pathway [23]. Low dose LPS preconditioning increased interferon regulatoryFactor 3 (IRF3) activity, upregulated anti-inflammatory/type I IFN gene expression, and suppressed nuclear factor kappa B (NF-κB) activity to reduce ischemic injury and improve cell survival. A clinical study (centrality value 0.13) focused on haemodynamic and metabolic changes mediated by RIPC in patients with subarachnoid hemorrhage (SAH) [24]. The results demonstrated that metabolic effects played a more durable role in ischemic protection for 24–54 h after RIPC. The latest article with a high centrality value (0.14) published by Liu ZJ in 2016 revealed the significant changes in peripheral immune responses to stroke after repeated RICP [25]. Peripheral immune changes during RIPC-mediated neuroprotection against stroke mainly included suppression of T-cell (CD3^+^ CD8^+^) exudation, blocking the reduction of NKT cells (CD3^+^/CD161a^+^), reversal of the reduced B-cell population, and elevation of noninflammatory monocytes, interleukin-6 (IL-6) and tumour necrosis factor α（TNFα）.

**Table 5:** Top 7 cocited references with the highest centrality.

Rank	Co-cited reference	Centrality	Frequency	Impact Factor	Type
1	Ren C, 2008, NEUROSCIENCE, https://doi.org/10.1016/j.neuroscience.2007.11.056	0.39	22	3.3	Article
2	Meller R, 2005, J CEREBR BLOOD F MET, https://doi.org/10.1038/sj.jcbfm.9600024	0.34	21	6.3	Article
3	Gonzalez NR, 2013, ACTA NEUROCHIR SUPPL, https://doi.org/10.1007/978-3-7091-1192-5_36	0.16	14	NOT FOUND	Article
4	Liu ZJ, 2016, CNS NEUROSCI THER, https://doi.org/10.1111/cns.12448	0.14	23	5.5	Article
5	Marsh BJ, 2009, NEUROSCIENCE, https://doi.org/10.1016/j.neuroscience.2008.07.067	0.13	11	3.3	Review
6	Shimizu S, 2001, J CEREBR BLOOD F MET, https://doi.org/10.1097/00004647-200103000-00007	0.11	22	6.3	Article
7	Vartanian KB, 2011, J NEUROINFLAMM, https://doi.org/10.1186/1742-2094-8-140	0.10	14	9.3	Article

### 3.6 Analysis of clusters and timeline maps of cocited references

By using keywords extracted from the cocited literature and the log-likelihood ratio (LLR) algorithm, a cluster network map was generated (Fig. 4 B). Q = 0.766 > 0. 3, S = 0.887 > 0.7, suggesting that the cluster structure generated was significant and reliable. Each cluster contained some terms, the first one of which represented the cluster label. The top 11 of the 22 clusters are shown in Fig. 4 (B) and Table 6, including: #0 tolerance, #1 hif-1 alpha, #2 perconditioning, #3 cyclooxygenase-2, #4 erythropoietin, #5 diazoxide, #6 hypoxic preconditioning, #7 normobaric hyperoxia, #8 Toll-like receptors, #9 postconditioning, and #10 secretome. Different clusters are involved in different types of preconditioning and molecular mechanisms of triggered neuroprotective effects.

**Table 6:** Top 11 clusters of cocited references.

Cluster ID	Size	Silhouette	Coverage	Label
0	201	0.878	tolerance; neuronal death; seizures; rats; glutamate receptors	tolerance
1	157	0.883	hif-1 alpha; meta-analysis; reperfusion injury; cerebral ischemic preconditioning; inflammatory cytokines	hif-1 alpha
2	150	0.802	perconditioning; autophagy; rapamycin; recurrence; hemorrhagic transformation	perconditioning
3	142	0.799	cyclooxygenase-2; organotypic slice culture; lps; adenosine a1 receptor; remote ischemic preconditioning	cyclooxygenase- 2
4	92	0.921	erythropoietin; protein expression; brain; hypoxic preconditioning (hpc); tnf-alpha	erythropoietin
5	72	0.95	diazoxide; volatile anesthetics; neuronal culture; mitochondria; glutamate	diazoxide
6	66	0.925	hypoxic preconditioning; hif; aging; collapsin response-mediated protein 2; peroxisome proliferator	hypoxic preconditioning
7	66	0.831	normobaric hyperoxia; brain ischemia tolerance; sevoflurane preconditioning; anesthetics glutathione reductase	normobaric hyperoxia
8	64	0.925	toll-like receptors; microrna; inflammation; tlr3; reprogramming	toll-like receptors
9	45	0.918	postconditioning; limb preconditioning; ischemic postconditioning; focal ischemia; post-conditioning	postconditioning
10	41	0.98	secretome; mesenchymal stem cells; mesenchymal stromal cells; cell therapy; neuroprotection	secretome

To further investigate the time distribution of the top 11 clusters, timeline maps were generated (Fig. 4C). Cluster labels on the right were sorted by the size of the clusters. The node on the timeline represents an article within that cluster. The time points in which the different nodes were located represented the time when the articles were first published. The solid timelines showed that these extracted terms were the hot topics of the period. As shown in Fig. 4 (C), clusters #0, #3, #4, and #5 were at the earliest stage. Clusters #2, #6, #7, #8, and #9 were in the middle stage. Clusters #1 and #10 were at the latest stage, reflecting the latest hot topics. Cluster #1 covered hif-1 alpha, meta-analysis, reperfusion injury, cerebral ischemic preconditioning, and inflammatory cytokines. Cluster #10 contained secretome, mesenchymal stem cells, mesenchymal stromal cells, cell therapy, and neuroprotection.

### 3.7 Analysis of co-occurring keywords

**Fig. 5:**
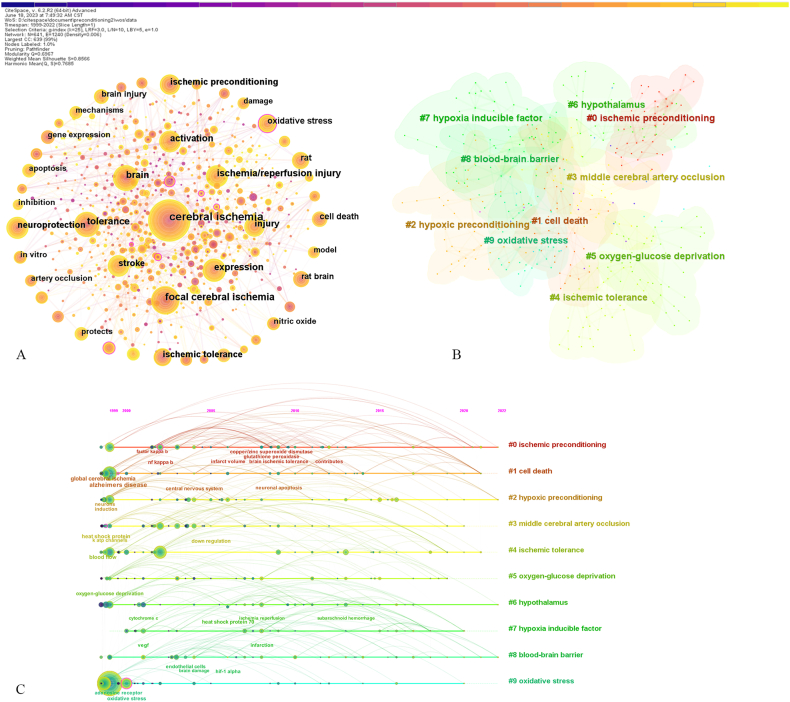
(A) The network of co-occurring keywords. (B) The cluster map of co-occurring keywords. (C) The timeline map of co-occurring keywords.

Keywords highly summarize the research topic of a scientific article. Study on the evolution of keywords helps to understand the trends of research hotspots in the field. After merging synonymous phrases and pruning the network (parameter selection: pathfinder and pruning the merged network), a keyword co-occurrence network map with 641 nodes and 1,240 links was generated (Fig. 5A). A total of 641 keywords met the selection criteria, 27 of which appeared over 100 times. The top 20 keywords are listed in Table 7, including cerebral ischemia (744 times), focal cerebral ischemia (372 times), tolerance (315 times), brain (295 times), expression (282 times), ischemia/reperfusion injury (250 times), activation (247 times), stroke (238 times), injury (229 times), neuroprotection (222 times), ischemic preconditioning (210 times), ischemic tolerance (208 times), oxidative stress (181 times), cell death (170 times), rat (169 times), rat brain (164 times), brain injury (147 times), nitric oxide (124 times), apoptosis (123 times) and gene expression (121 times).

**Table 7:** Top 20 keywords in frequency and top 7 keywords in centrality.

Rank	Keywords	Frequency	Centrality	Rank	Keywords	Centrality	Frequency
1	cerebral ischemia	744	0	1	channels	0.17	18
2	focal cerebral ischemia	372	0.01	2	Alzheimer's disease	0.13	9
3	tolerance	315	0.01	3	oxidative stress	0.11	181
4	brain	295	0.01	4	NF kappa b	0.11	77
5	expression	282	0.01	5	cell survival	0.11	6
6	ischemia/reperfusion injury	250	0.06	6	heat shock protein	0.1	36
7	activation	247	0.04	7	neurotrophic factor	0.1	20
8	stroke	238	0.02				
9	injury	229	0.01				
10	neuroprotection	222	0.01				
11	ischemic preconditioning	210	0.02				
12	ischemic tolerance	208	0				
13	oxidative stress	181	0.11				
14	cell death	170	0.02				
15	rat	169	0.02				
16	rat brain	164	0.01				
17	brain injury	147	0.07				
18	nitric oxide	124	0.03				
19	apoptosis	123	0.08				
20	gene expression	121	0.05				

Keywords with high centrality values (≥0.1) are displayed in Table 7, including channels (0.17), Alzheimer's disease (0.13), oxidative stress (0.11), NF kappa b (0.11), cell survival (0.11), heat shock protein (0.10) and neurotrophic factor (0.10).

These hot keywords mainly covered related cerebrovascular diseases, types of preconditioning, major animal models, strategies for neuroprotection against injury, molecular mechanisms of ischemic tolerance, and critical factors.

### 3.8 Analysis of keyword clustering and timeline

A keyword clustering network map was generated by using the LLR algorithm. Q = 0.697 > 0. 3, S = 0.857 > 0.7, meeting a valid clustering. A total of 19 clusters were formed, the largest 10 of which are shown in Fig. 5 (B) and Table 8. Each cluster was made up of 5 typical keywords, and the most representative keyword was selected as the cluster label. These clusters were summarized as the following research hotspots.

**Table 8:** Top 10 keywords clusters.

Cluster ID	Size	Silhouette	Coverage	Label
0	42	0.839	ischemic preconditioning; nNOS; superoxide dismutase; normobaric hyperoxia; brain ischemia tolerance	ischemic preconditioning
1	42	0.811	cell death; global cerebral ischemia; neuronal apoptosis; map kinase; neuronal preconditioning	cell death
2	42	0.861	hypoxic preconditioning; mesenchymal stem cells; protein expression; induction; endoplasmic reticulum stress	hypoxic preconditioning
3	40	0.789	rrid; middle cerebral artery occlusion; methyl d aspartate; metallothionein; transient middle cerebral artery occlusion	middle cerebral artery occlusion
4	40	0.911	ischemic tolerance; remote ischemic preconditioning; toll-like receptors; transient ischemia; tnf alpha	ischemic tolerance
5	37	0.885	oxygen-glucose deprivation; transporters; cerebral ischemia; transplantation; cellular engineering	oxygen-glucose deprivation
6	35	0.83	hypothalamus; glutamate receptors; neuronal death; cytochrome *c*; brain edema	hypothalamus
7	35	0.819	hypoxia inducible factor; endothelial growth factor; trans resveratrol; ischemic cerebrovascular disease; factor i	hypoxia inducible factor
8	34	0.817	blood-brain barrier; inducible factor i; hif-1 alpha; epo; metabolites	blood-brain barrier
9	34	0.881	oxidative stress; cerebral ischemia; expression; protein kinase c; programmed cell death	oxidative stress

#### 3.8.1 Ischemic neuronal injury and death, and related pathological mechanisms

Clusters #1, #6, and #9. Associated keywords mainly include cerebral ischemia, brain edema, cell death, and neuronal death. Mechanisms are involved in oxidative stress, excitotoxicity, apoptosis, and other forms of programmed cell death.

#### 3.8.2 Types of preconditioning

Clusters #0, #2 and #4. Ischemic preconditioning (IPC) and hypoxic preconditioning (HPC) are the two most common methods of inducing ischemic tolerance. Remote ischemic preconditioning, as well as local ischemic preconditioning, is a member of the ischemic preconditioning family. TNFα is an essential cytokine capable of inducing ischemic tolerance. Some critical enzymes and receptors, such as neuronal nitric oxide synthase (nNOS), superoxide dismutase (SOD), and TLRs, mediate the generation of ischemic tolerance.

#### 3.8.3 Methods of preconditioning model

Clusters #3 and #5. Middle cerebral artery occlusion is a common and typical model of focal cerebral ischemia in vivo, while in vitro the OGD model is chosen to mimic cellular ischemia.

#### 3.8.4 Key factors in neuroprotection

#7 and #8. Hypoxia-inducible Factor 1 α (HIF1α), a key regulator of ischemic tolerance, regulates the expression of various genes, including erythropoietin (EPO) and vascular endothelial growth factor (VEGF). EPO and VEGF participate in cell survival and neuronal repair. Maintaining the integrity of the blood-brain barrier is essential to reduce ischemic injury.

Timeline maps (Fig. 5C) were established to further explore the temporal distribution of these clusters. Each cluster was represented by a horizontal timeline, arranged from top to bottom by size, with the label listed on the right. The nodes on the same timeline represented keywords in the same cluster. The solid timeline reflected the active time span of the cluster. As shown in Fig. 5 (C), all the clusters spanned a long time period, which almost encompassed the duration of our study. Such results indicate that the research topics represented by these clusters have been of long-term interest to researchers and are important parts of the knowledge base in the field of preconditioning.

### 3.9 Analysis of keywords with citation bursts

**Fig. 6:**
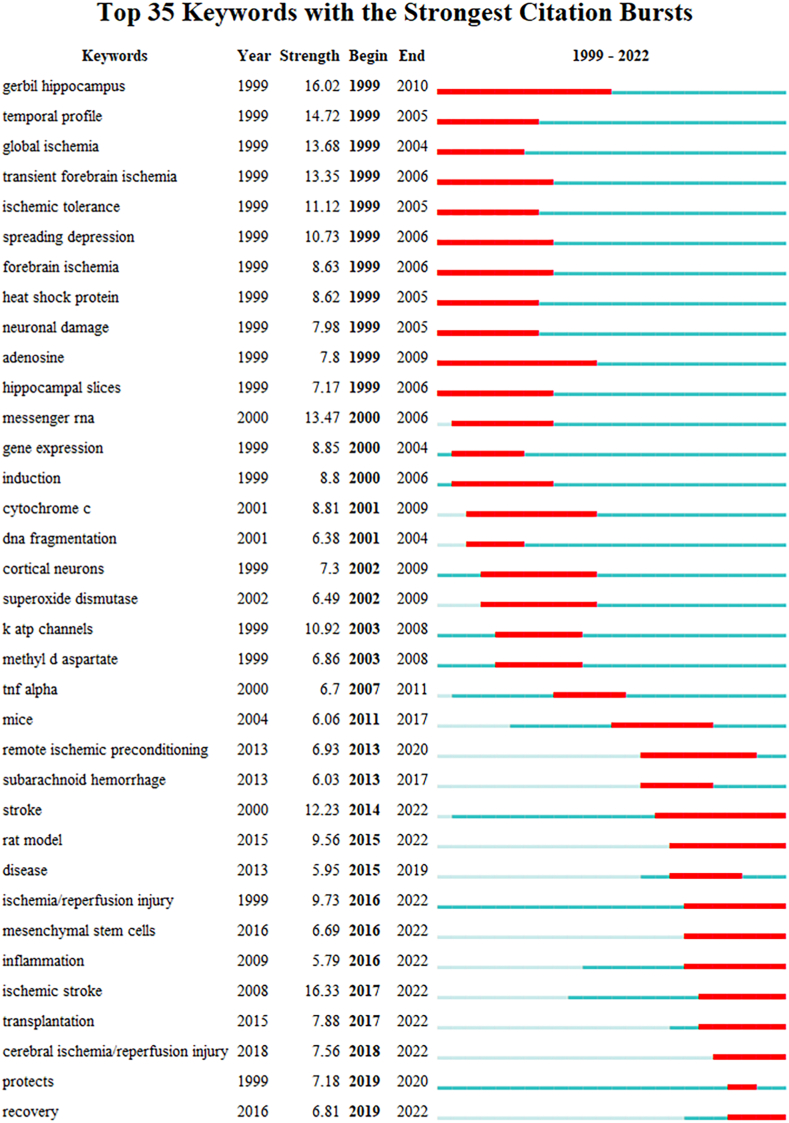
Top 35 keywords with the strongest citation bursts.

Briefly, keywords with citation burst refer to keywords that have been highly cited in a short period of time. Burst detection enables the temporal location of burst keywords to explore hotspot trends and research frontiers. The top 35 keywords with the strongest citation bursts are shown in Fig. 6. The red line represents the duration of the keyword burst. In summary, the research hotspots were divided into two stages with 2012 as the demarcation point. The differences between these two stages were as follows：

#### 3.9.1 Neuroprotection strategies

Generally, the neuroprotective effects of preconditioning are manifested in two ways: one is to reduce ischemia or reperfusion injury, and the other is to repair and regenerate. The burst keyword "neuronal damage" implied that the researchers had highlighted the neuroprotection of ischemic preconditioning and ischemic tolerance against neuronal damage and death before 2012. However, neuronal repair and regeneration have received increasing attention in recent years, supported by the burst keywords "recovery", "transplantation" and "mesenchymal stem cells". The role of stem cell transplantation in repairing brain damage is an emerging research hotspot.

#### 3.9.2 Types of preconditioning

Prior to 2012, researchers conducted extensive studies on preconditioning, exploring a range of stimuli without highlighting any type of preconditioning. With increasing emphasis on safety in recent years, researchers have devoted more attention to RIPC, especially noninvasive limb ischemic preconditioning. In addition, stem cells can be used as therapeutic tools to repair brain damage. Notably, preconditioning of cultured stem cells for transplantation can enhance the therapeutic effect. Unlike conventional preconditioning, this novel type of preconditioning is aimed at therapeutic tools (stem cells cultured in vitro), rather than at stroke individuals, effectively avoiding the uncertain risks to individuals from preconditioning. This indirect preconditioning is an emerging research hotspot.

#### 3.9.3 Cerebral ischemia animal model

The gerbil model received a great deal of interest from researchers before 2010. The explanation might be that gerbils have special anatomy. The lack of posterior communicating artery branches prevents the internal carotid artery system and the vertebrobasilar artery system from being connected. An ideal model of forebrain ischemia can be easily created by blocking the bilateral carotid arteries. With advances in technology and materials, the suture-occluded method has shown advantages in local cerebral ischemia models. An increasing number of researchers are using mouse and rat models to establish MCAO models.

#### 3.9.4 Mechanisms

Burst keywords related to the mechanisms in the early stage included "heat shock protein", "adenosine", "messenger RNA", "gene expression", "cytochrome *c*", "superoxide dismutase", "methyl d aspartate", "K atp channels" and "TNFα". These keywords were strong evidence of the importance that researchers place on preconditioning mechanisms. Neuroprotection at the cellular and molecular levels mainly included anti-apoptosis, antioxidative stress, anti-cytotoxicity, anti-inflammation, correction of ionic disorders, suppression of energy metabolism, and regulation of gene expression. In recent years, anti-inflammation has been refocused on.

## 4 Discussion

### 4.1 General information

In our study, CiteSpace was used to analyse 1,738 papers on preconditioning in cerebral ischemia from different dimensions between 1999 and 2022. The number of annual publications showed a trend of increasing and then decreasing, with 2013–2016 being the high point of research in the field. Research was on the rise until 2015 with enthusiasm rising. Different stimuli and mechanisms were the focus of attention during this period. Research enthusiasm calmed down after 2016, possibly related to research bottlenecks or poor clinical translation. However, there have been more than 55 articles published annually in this field since 2004, suggesting that the field has been receiving widespread attention from researchers and is still being studied in depth.

In general, the United States is the most active collaborator with other countries and contributes the most to the field. There are several reasons for this: First, the US is not only the country with the most publications, but also the one with the largest centrality value. Second, four of the top ten institutions in terms of publications are from the US. The University of Miami contributes the second most studies, while Harvard University with the largest centrality value plays an important bridging role in institutional collaboration. Third, Perez-Pinzon, Miguel A, from the University of Miami, is the author with the highest number of publications. The above factors indicate that the United States is a productive and high-impact country in this field.

China is second to the US in terms of the number of publications. Despite its slow start, China has contributed almost half of the annual publication volume since 2016, showing a burgeoning trend, and indicating that the field has attracted significant interest from Chinese researchers. Four of the top ten institutions in terms of publications are from China, with Capital Medical University being the most prolific, suggesting that China also attaches importance to preconditioning research. However, the low centrality value implies a lack of cooperation with other countries. More cooperation between China and other countries or institutions needs to be strengthened in the future.

In addition, KITAGAWA K from Japan is the most cited author and the first to introduce cerebral ischemic tolerance, leading the way in this area of research. Following KITAGAWA are DIRNAGL U from Germany and GIDDAY JM from the USA, whose studies have been widely accepted. A research group from South Korea has published a large number of articles in this field since 2014.

### 4.2 Research hotspots in the knowledge base of preconditioning in cerebral ischemia

The cocitation reference network and co-occurrence keyword network provide us with a wide range of knowledge on preconditioning in cerebral ischemia. Through the analysis of keyword frequency, centrality, clustering, and integration of key references, we summarized three research hotspots in the context of relevant knowledge, including (1) relevant diseases and experimental models (2) types of preconditioning and stimuli, and (3) mechanisms of ischemic tolerance.

#### 4.2.1 Relevant diseases and experimental models

Preconditioning is emphasized in the investigation of several brain diseases, such as ischemic cerebrovascular disease, neonatal hypoxic ischemia encephalopathy (NHIE), delayed cerebral ischemia after subarachnoid hemorrhage, surgery or trauma-related brain injury, and neurodegenerative diseases. Based on keyword analysis, ischemic cerebrovascular diseases were found to be the primary hot research diseases, including ischemic stroke and transient cerebral ischemia. Ischemic stroke is a serious health threat to elderly individuals, with high mortality and disability rates [1]. Ischemia/reperfusion (I/R) injury is the predominant pathological injury. Transient ischemic attack (TIA) can trigger ischemic tolerance without lethal injury to brain tissue, and is regarded as ischemic preconditioning in humans [26].

Animal models of cerebral ischemia mainly include global and focal cerebral ischemia. Focal cerebral ischemia has a higher frequency in keyword analysis, indicating a hot spot for research. The region supplied by the middle cerebral artery (MCA) is the most common location for ischemic stroke in humans. Therefore, MCAO is the primary model of focal cerebral ischemia [27]. The intraluminal suture occlusion method is commonly used for MCAO due to its ease of performance, high success rate, and ability to allow for ischemic reperfusion [28]. A surgical nylon filament with a round tip was inserted into the internal carotid artery (ICA) through the common carotid artery (CCA) or external carotid artery (ECA). The MCA is occluded when the round tip reaches the bifurcation of the MCA and is reperfused when the round tip withdraws below the bifurcation. Global cerebral ischemia simulates brain damage during human cardiac arrest/resuscitation, mainly achieved by large vessel occlusion and cardiac arrest [29].

For the selection of animals, rodents such as rats, mice, and gerbils are the most popular species used [28]. Rats are the most commonly used animal model due to their low cost, cerebrovascular structure similar to that of humans, and excellent experimental reproducibility; this was supported by our findings, with the highest frequency in rats. The advantages of mice are easy acquisition and genetic modification [30]. Gerbils are often chosen as models of forebrain ischemia because of the incomplete circle of Willis [31].

The most widely used model in vitro is oxygen-glucose deprivation (OGD) to simulate ischemic stroke [32]. A brief period of OGD can simulate ischemic preconditioning, while a longer period of OGD followed by the restoration of oxygen and glucose supply can simulate ischemia/reperfusion. It was shown that cultured cells after preconditioning had lower mortality and higher cell activity, suggesting the induction of ischemic tolerance. In addition, hypoxic preconditioning and pharmacological preconditioning are often chosen to trigger ischemic tolerance [33,34].

#### 4.2.2 Types of preconditioning and stimuli

In recent decades, researchers have studied a wide variety of endogenous or exogenous stimuli. Preconditioning can be divided into different types, based on different harmful stimuli such as ischemia, hypoxia, pharmacological substances, hyperbaric oxygen, abnormal temperature, chemical substances, exercise stress, and electroacupuncture [8]. Ischemic preconditioning had the highest frequency and the largest cluster among them in our study, followed by hypoxic preconditioning with a smaller cluster and finally volatile anaesthetic preconditioning with the smallest cluster (#14 in keyword clusters, not shown in Fig. 5B and Table 8). These three types are the focus of preconditioning research.

##### 4.2.2.1 Ischemic preconditioning (IPC)

Ischemic preconditioning is the most classic model and includes local ischemic preconditioning and remote ischemic preconditioning. Local ischemic preconditioning is aimed at the large arteries supplying blood to the brain, covering focal cerebral ischemia and global cerebral ischemia. Numerous studies have confirmed the reduction in infarct size and improvement in neurological deficits mediated by local ischemic preconditioning. Both focal cerebral ischemia and global cerebral ischemia can be used as triggers, but the duration of exposure varies. Exposure to focal cerebral ischemia typically lasts 10–20 min, and in some cases up to 30 min [35]. Single local ischemic preconditioning of fewer than 2 min does not induce ischemic tolerance [8]. Exposure to global cerebral ischemia is commonly 2–3 min, and more than 5 min can lead to permanent brain damage. TIA is a common ischemic cerebrovascular disease, consistent with endogenous local ischemic preconditioning. Evidence has shown that stroke patients with prior TIA have a better prognosis than those without TIA, indicating that TIA induces ischemic tolerance [26,[36], [37], [38], [39]]. It was supported by a systematic review and meta-analysis, revealing that patients with prior TIA had lower NIHSS scores at admission and lower MRS scores on discharge [39]. However, there are different findings. In a retrospective cohort study of 3,530 ischemic stroke patients from Switzerland, researchers found that although prior ischemic events significantly reduced the hospital admission severity in stroke patients, there was no positive effect on early stroke recovery and 3-month prognosis [40]. Several other studies also failed to confirm that TIA improved subsequent stroke outcomes [[41], [42], [43]]. This may be related to individual differences in age, underlying cerebrovascular status, etc. Remote ischemic preconditioning refers to repeated transient occlusion of other parts of the body, which is later described in detail.

##### 4.2.2.2 Hypoxic preconditioning (HPC)

As an oxygen-demanding organ of the body, the brain is highly sensitive to changes in oxygen concentration. Under hypoxic (usually 8 %) conditions, the brain undergoes sublethal insults that induce tolerance to subsequent lethal damage. Hypoxic preconditioning has been extensively studied in ischemic stroke and neonatal hypoxic ischemia encephalopathy. In vivo and in vitro studies have demonstrated the induction of ischemic tolerance in neurons by HPC [4,44,45]. Exposure to hypoxic preconditioning with 8 % O_2_ for 30 min downregulated neuronal IL-1R1 expression and reduced the phosphorylation level of mixed lineage kinase domain-like (MLKL), thereby attenuating neuronal necroptosis after fatal global cerebral ischemia [46]. In an in vitro study, researchers established a human neuronal model simulating the ischemic penumbra and found that hypoxic preconditioning attenuated neural damage from a second hypoxia, possibly related to enhanced neuronal functional connectivity [34].

##### 4.2.2.3 Volatile anaesthetics preconditioning

In vivo and in vitro studies have shown that inhalation anesthetics preconditioning, including isoflurane and sevoflurane, can induce ischemic tolerance against I/R injury [[47], [48], [49], [50]]. There has been more research on sevoflurane preconditioning in recent years. Preexposure to sevoflurane provides neuroprotection in models of global ischemia [51], focal ischemia [52], and neonatal brain injury [53,54]. In a focal cerebral ischemia model, sevoflurane preconditioning reduced cerebral infarct size, improved neural function, and reduced apoptosis through anti-inflammation (e.g., by reducing tumour necrosis factor-alpha and interleukin 1-β (IL1-β) levels) [55]. Further studies showed that inhibition of pro-inflammatory factors and promotion of anti-inflammatory mediators were mediated by GSK-3β (glycogen synthesis kinase-3β) -dependent Nrf2 (nuclear factor erythroid 2-related Factor 2). Knockout of Nrf2 abolishes this neuroprotection [56].

#### 4.2.3 Mechanisms of ischemic tolerance

Cellular damage resulting from ischemia/reperfusion is a multifaceted process that involves various pathological mechanisms, including energy depletion, ionic imbalances (such as calcium overload), excitotoxicity, oxidative stress, inflammation, and apoptosis. These factors ultimately lead to cell death.

This pathological process involves, but not limited to, the following signalling pathways: **NMDA receptor pathway**: I/R injury leads to excessive glutamate release and overactivation of NMDA receptors, triggering calcium influx, oxidative stress, and cell death [57]. **Mitochondria-dependent apoptotic pathway**: I/R injury triggers a decrease in mitochondrial membrane potential and changes in mitochondrial membrane permeability, which releases apoptosis-associated proteins (e.g., cytochrome C, apoptosis-inducing factors) into the cytoplasm and activates the caspase family of enzymes, leading to apoptotic cell death [58]. **TLR signalling pathway**: Activation of TLR family members activates the transcription factor NF-κB and the regulatory factor AP-1, leading to the production of inflammatory mediators and an enhanced inflammatory response [22,59]. **NF-κB pathway**: I/R activates NF-κB, which promotes the release of inflammatory mediators and the expression of cell adhesion molecules, leading to inflammatory responses and cell injury [60,61]. **Mitogen-activated protein kinase (MAPK) signalling pathway**: This pathway includes kinases such as p38 MAPK, JNK (c-Jun N-terminal Kinase) and ERK (extracellular signal-regulated kinase). MAPK signals by phosphorylating a range of downstream proteins, including transcription factors and apoptosis-associated proteins. ERK is commonly associated with cell survival, whereas JNK and p38 MAPK are commonly associated with cell death and inflammatory responses [62]. **Phosphatidylinositol-3 kinase/protein kinase B (PI3K/Akt) signalling pathway**: In I/R, activated PI3K kinase phosphorylates Akt, to activate downstream signalling cascades, such as inhibiting the activity of apoptosis-related proteins or promoting the expression of cell survival-related proteins, to exert a protective effect [63,64]. **Nrf2-ARE** (antioxidant response elements) **pathway**: Under oxidative stress conditions, Nrf2 is released and translocates into the nucleus, where it binds to ARE to initiate the transcription of antioxidant genes, including glutathione peroxidase (GSH-PX), heme oxygenase (HO-1), SOD and glutathione reductase (GR), to enhance cellular antioxidant effects [65,66]. These mechanisms are part of the complex network of signalling pathways. Although various preconditioning methods involve large and complex cascading networks, the ultimate targets of action fall on the above damage mechanisms. Therefore, neuroprotection against ischemia/reperfusion injury is common to different preconditioning methods. We summarized and listed the top 3 ischemic tolerance mechanisms in keywords and references analysis **(**Table 9**). Oxidative hypoxia-inducible Factor 1, and ion channels are the top concerns of researchers.**

**Table 9:** Top 3 mechanisms in keyword and reference analysis.

Rank	Keywords (frequency)	Keywords (centrality)	Keywords Clusters (size)	References Clusters (size)
1	oxidative stress (181)	channels (0.17)	hypoxia inducible factor (35)	hif-1 alpha (157)
2	nitric oxide (124)	oxidative stress (0.11)	blood-brain barrier (34)	cyclooxygenase 2 (142)
3	apoptosis (123)	NF kappa b (0.11)	oxidative stress (34)	erythropoietin (92)

##### 4.2.3.1 Oxidative stress

As brain tissue is one of the organs with the highest energy requirements, reactive oxygen species (ROS) are constantly produced even under normal physiological conditions, including free radicals (e.g., superoxide anion radicals, hydroxyl radicals and peroxyl radicals), hydrogen peroxide, and monoclinic oxygen [67,68]. A powerful endogenous antioxidant system consisting of antioxidant enzymes and low molecular antioxidants neutralizes reactive oxygen species to maintain a balance between oxidation and antioxidation. Under pathological conditions, the excessive production and reduced removal of ROS lead to continuous accumulation, damaging neuronal structures and even inflicting cell death.

In ischemia, glutamate excitotoxicity, calcium overload, and mitochondrial dysfunction are involved in ROS generation [58,69]. After arterial occlusion, energy depletion leads to decreased Na^+^/K^+^ ATPase pump activity, depolarization of nerve cell membranes, and opening of voltage-dependent Ca^2+^ channels, with the resultant Ca^2+^ influx and excessive glutamate release [70]. Excess glutamate in the synaptic gap activates NMDAR and AMPAR (amino-3-hydroxy-5-methyl-4-isoxazole propionate receptor) leading to significant Ca^2+^ influx. Final cytoplasmic calcium overload occurs as a result of further enhancement of Ca^2+^ influx, reduction of calcium pump activity, and release of endoplasmic reticulum calcium [71,72]. Intracellular calcium overload transfers into mitochondria via voltage-dependent anion channels (VDAC), resulting in mitochondrial calcium overload and opening of the mitochondrial permeability transition pore (MPTP) [73]. Altered mitochondrial membrane permeability leads to mitochondrial injury, dysfunction, increased ROS production and cytochrome *c* release [74]. The generated ROS further exacerbated mitochondrial injury. In the cytoplasm, cytochrome *c*, apoptosis protease activating Factor 1 (APAF1), and procaspase-9 bind to form apoptosomes, leading to the initiation of apoptosis [58]. In reperfusion, the inhibited mitochondrial respiratory chain resets in a short period of time, leading to a large increase in superoxide anion radicals [[75], [76], [77]]. Neuronal NADPH oxidase (NOX) and xanthine oxidase activities are also significantly increased, producing large amounts of ROS [78,79].

Excess ROS directly oxidize DNA, causing DNA double-strand breaks and structural abnormalities; ROS contribute to lipid peroxidation, changing the fluidity and permeability of cell or organelle membranes; and ROS denature or cleave proteins, affecting the structure and physiological function of various proteins or enzymes [58]. ROS disrupt the blood-brain barrier and promote inflammatory responses and apoptosis [77].

Cellular antioxidant strategies include the following three main types: (1) reducing ROS production, (2) neutralizing ROS through antioxidant enzymes and antioxidants, and (3) repairing oxidative damage to key biomolecules such as DNA, proteins, and lipids [80]. Antioxidants include glutathione, vitamin C, bilirubin, melatonin, and uric acid [81]. Antioxidant enzymes and proteins include SOD, catalase (CAT), GSH-PX, glutathione reductase, HO-1 and peroxiredoxin-1 (Prx-1) [67]. HO-1 is an important antioxidant enzyme, and among its downstream products, CO is vasodilating, anti-apoptotic, and anti-inflammatory [82], whereas biliverdin and bilirubin are considered to be antioxidant and anti-inflammatory [83]. Reperfusion injury induces an increase in the transcriptional activity of the HO-1 gene and an increase in the synthesis and expression of the HO-1 protein, which is mainly regulated by Nrf2 [84,85]. During reperfusion, Prx-1 expression is significantly increased in brain cells [86,87]. On the one hand, Prx-1 plays an antioxidant role by downregulating the level of hydrogen peroxide, attenuating ischemia-reperfusion injury [88,89]. Ubiquitination of Prx 1 worsens brain tissue injury [90]. On the other hand, brain cells release Prx-1, which has a potent activating effect on macrophages, leading to a harmful inflammatory cytokine response that promotes neuronal cell death [91,92].

Preconditioning enhances the antioxidant capacity to resist neuronal damage, while ROS scavengers abolish preconditioning-induced neuroprotection [93]. Hyperbaric oxygen preconditioning upregulated HO-1 expression to attenuate oxidative stress injury in spinal cord neurons cultured in vitro [94]. Ischemic preconditioning attenuated cerebral I/R injury in rats by promoting HO-1 expression in the brain [95]. Ischemic preconditioning increases SOD2 and reduces superoxide anion radical production by increasing the expression of peroxynitrite 2 to exert antioxidant effects, which is abolished by thioredoxin 2 inhibitors [96]. Preconditioning exercise alleviated neurological dysfunction and cognitive impairment in rats with cerebral ischemia, possibly associated with increased antioxidant capacity of neurons in the hippocampal CA1 region, due to the detection of increased Klotho and MnSOD expression [97]. Electroacupuncture preconditioning reduced consumption of glutathione (GSH) and glutathione peroxidase-4 (GPX4) to attenuate oxidative stress injury after ischemia/reperfusion [98].

Antioxidant enzymes and low molecular antioxidants are regulated by Nrf2, which is inhibited in neurons [10]. The Keap1-Nrf2/ARE signalling pathway plays an important role [65]. Physiologically, Nrf2 is inactivated by Keap1 binding. Under oxidative stress, excess ROS prompts Nrf2 dissociation, activation, nuclear translocation, and binding to the ARE, which promotes the activation of downstream antioxidant enzymes (e.g., HO-1) for transcription and antioxidant effects [99]. The upstream PI3K/Akt pathway or MAPK signalling pathway can activate Nrf2/ARE signaling pathway and upregulate the expression of antioxidant enzymes [62,100]. Preconditioning with hyperbaric oxygen or hydrogen sulfide (H_2_S) can activate the PI3K/Akt/Nrf2/HO-1 antioxidant signalling pathway to attenuate ischemia/reperfusion injury [101,102]. Isoflurane preconditioning promoted Nrf2 expression and nuclear translocation, reversed the decrease in SOD and glutathione peroxidase (GSH-Px), and lowered the increase in malondialdehyde (MDA) to protect cortical neurons [33]. Nrf2-mediated neuroprotection was also observed in other preconditioning models [103,104]. As Nrf2 is highly expressed in astrocytes, astrocytes provide powerful antioxidant support for neurons. Neurons can absorb GSH precursors supplied by astrocytes to synthesize their own GSH for antioxidant action. IPC activated Nrf2 in astrocytes and thereby increased the expression of downstream antioxidant products to induce ischemic tolerance, while knockout of Nrf2 significantly reduced the neuroprotective effect [105,106].

##### 4.2.3.2 Hypoxia-inducible factor 1

Hypoxia-inducible Factor 1(HIF-1) is a key transcription factor in the brain's response to hypoxia, of which the α subunit is oxygen concentration sensitive. In the presence of sufficient oxygen, the α subunit is not stable owing to ubiquitination or degradation. Under hypoxic conditions, HIF-1α is significantly upregulated and stably present, combining with the β subunit to form dimers involved in regulating the expression of various downstream genes such as EPO [107], VEGF [108], glucose transporters (GLUTs) [109], and glycolytic enzymes [110]. HIF-1α plays a key role in the induction of ischemic tolerance by preconditioning [[111], [112], [113]]. The preconditioning-induced increase in HIF-1α is differentiated between neurons and astrocytes, being hypoxia dependent, rapid, and transient (1–3 days) in neurons and P2X7-mediated, slow, and persistent (3 days - at least 2 weeks) in astrocytes [114,115].

In gerbil forebrain ischemia models, 2-min ischemic preconditioning reduced neuronal death in the CA1 region of the hippocampus [108]. This neuroprotective effect was mediated by HIF-1α, which was abolished by a HIF-1α inhibitor. In neonatal ischemic-hypoxia models, HIF-1α was the critical factor for HPC to reduce brain damage [116]. Elevated HIF-1α enhanced the expression of VEGF, while no protective effect of HPC against ischemic hypoxia was observed in HIF-1α knockout mice [117]. In rat traumatic brain injury (TBI) models, HPC preconditioning attenuated cell injury and death by a mechanism related to HIF-mediated enhancement of glucose transport activity [109]. A 3-week preconditioning exercise induced ischemic tolerance through upregulation of HIF-1α expression [118]. Further studies revealed that high expression of HIF-1α for two weeks was mediated by astrocyte P2X7 receptors, demonstrating that astrocytes are involved in durable cerebral ischemic tolerance through upregulation of HIF-1α [119].

##### 4.2.3.3 Ion channels, exchangers, and homeostasis

Cellular ionic homeostasis is essential for neurons. Both ion channels and exchangers are proteins in biological membranes that enable the transport of charged ions across the membrane and regulate extra and intracellular ion concentrations. During cerebral ischemia and reperfusion, abnormal opening and closing of ion channels cause K+ outflow and Na^+^, Ca^+^, and Cl^−^ inflow, resulting in disturbed ion balance intra and extracellularly, in particular calcium overload, ultimately damaging neurons [120]. Preconditioning can prevent ischemic injury by regulating the expression and activation of channel proteins to maintain cellular ion homeostasis.

Researchers previously investigated K_ATP_ channels earlier, which are divided into mitochondrial K_ATP_ (mitoK_ATP_) and sarcolemma K_ATP_ (sarcK_ATP_). Studies show that mitoK_ATP_ channel expression and activation play a crucial role in the ischemic tolerance induced by preconditioning [121,122]. The mitoKATP-selective inhibitor 5-HD eliminates ischemic preconditioning-mediated neuroprotection [123], while the openers diazoxide and BMS-191095 induce and enhance tolerance to brain injury [[124], [125], [126], [127], [128]].

Another group of proteins that play a key role in the development of ischemic injury are the Na^+^/Ca^2+^ exchangers (NCXs) in neuronal membranes, including NCX1, NCX2, and NCX3, which maintain ionic homeostasis by regulating Na^+^ and Ca^2+^ [120]. The important role of NCX1 and NCX3 in ischemic tolerance has been identified both in vitro and in vivo for a long time [[129], [130], [131], [132]]. Preconditioning induced an increase in NCX1 and NCX3 expression, while the absence of NCX1 and NCX3 prevented neuroprotection [133]. A recent study showed that in focal cerebral ischemia models, ischemic preconditioning upregulated the expression of K^+^-dependent Na^+^/Ca^2+^ exchanger isoform 2 (NCKX2), while knockout of NCKX2 significantly prevented the preconditioning-induced reduction in infarct volume and improvement in neurological function, demonstrating that NCKX2 is also essential for neuroprotection induced by preconditioning [134].

In addition, some other channels are involved in preconditioning neuroprotection. In research exploring the mechanisms of volatile anaesthetic preconditioning, it was discovered that neuroprotection induced by isoflurane and sevoflurane preconditioning was linked to the activation of the two-pore domain K^+^ channels TREK-2 and TREK-1, respectively [135,136]. In models of focal cerebral I/R and OGD/R, ischemic preconditioning promoted cell survival by inhibiting I/R-induced reduction of the ER (endoplasmic reticulum)-located calcium sensor STIM1 (stromal interacting molecule 1) and plasma membrane channel ORAI1, blocked by silencing of STIM1 or ORAI1 [35]. Moderate ethanol preconditioning reduced apoptosis elicited by OGD/R or I/R by elevating the expression of large conductance calcium-activated potassium channels (BK_Ca_) [137,138].

### 4.3 Emerging hotspots and research frontiers of preconditioning in cerebral ischemia

The analysis of temporal location, such as reference or keyword timeline maps and keyword burst analysis, allows us to identify emerging hot spots and research frontiers in the near future. The terms "mesenchymal stem cells", "transplantation" and "recovery" in the keyword burst analysis all point to stem cell transplantation. "Remote ischemic preconditioning" and "inflammation" are also recent flashpoints. The cluster #10 secretome in the reference timeline map (Fig. 4C) is the emerging cluster, in which the keywords include "secretome", "mesenchymal stem cells", "mesenchymal stromal cells", "cell therapy", and "neuroprotection". Taken together, combined with the research context, the emerging hotspots and research frontiers are remote ischemic preconditioning, preconditioning of mesenchymal stem cells (MSCs), and inflammation. The prominence of noninvasive RIPC and preconditioning of MSCs suggests that researchers are increasingly favour preclinical studies that are safe, effective, and that possess high potential for clinical application.

#### 4.3.1 Remote ischemic preconditioning (RIPC)

RIPC was first introduced in 1993 as a new ischemic preconditioning [139]. RIPC induces ischemic tolerance in target organs against lethal injury through repeated transient ischemia/reperfusion cycles in remote organs or tissues. RIPC has many advantages over local ischemic preconditioning, such as more triggers, simpler operation, greater safety, less pain, and easier access to clinical studies. Although studies have shown that preconditioning of many organs or tissues (e.g., liver [140], kidney [141]) successfully induces cerebral ischemic tolerance, noninvasive limb ischemic preconditioning (LIPC) is currently the most popular based on safety and practicality. LIPC usually involves brief cuff inflation to block blood flow and deflation to reperfuse. The most widely used protocol is currently 3 or 4 cycles of 5-min ischemia at 200 mmHg (or 20 mmHg over systolic pressure) [142]. Numerous animal studies have proven the neuroprotective effects of LIPC against ischemic stroke. For instance, RICP significantly enhanced cellular autophagy, decreased inflammatory response, and apoptosis to reduce neurological deficit scores and infarct size in a rat focal ischemia/reperfusion model, using 3 cycles of 5 min of ischemia and 5 min of reperfusion in the femoral artery [143]. Two meta-analyses on animal experiments also supported the powerful effect of remote ischemic conditioning (including RIPC) in reducing cerebral infarct volumes and improving prognosis [144,145].

Long-term administration of bilateral upper arm ischemic preconditioning (BAIPC) has shown positive effects on ischemic cerebrovascular disease, by 5 cycles of 5-min ischemia and 5-min reperfusion at 200 mmHg, twice or three times daily [143,146,147]. The treatment has been found to improve cerebral perfusion, promote anticoagulation, reduce platelet aggregation, and lower the risk of stroke and TIA recurrence rates. Short-term administration of LIPC reduces the incidence of new ischemic events after brain tumour surgery by 5 cycles of 5 min of ischemia and 5 min of reperfusion at 200 mmHg following anaesthesia [148]. In a small retrospective case-control study patients with peripheral vascular disease (PVD) had lower NIHSS scores at admission, MRS scores at discharge, infarct size, and mortality after stroke than those without PVD, suggesting that chronic ischemia in the limb may have induced cerebral ischemic tolerance [149]. Interestingly, in another large prospective study, researchers did not find a significant positive effect of PVD on ischemic stroke patients treated with endovascular thrombectomy (EVT) after correcting for possible confounders [150]. This may be explained by the fact that timely EVT has a much greater impact on ischemic stroke outcomes than RIPC, and further clarification is needed from more comprehensive and extensive studies.

RIPC successfully induces ischemic tolerance not only in the brain, but also in the heart [151], kidneys [152], lungs [153], and liver [154], which seems to suggest that repeated ischemic preconditioning of one artery may have induced ischemic tolerance throughout the body. How does this protective effect extend from local to other areas? It is believed that there are two major pathways, humoral and neural [142]. Researchers found that RIPC significantly altered the immune system in the peripheral blood prior to ischemic stroke [25,155]. Prostaglandins, brain-derived neurotrophic factor (BNDF), and VEGF were detected to be elevated in the blood after RIPC in a healthy population [156]. The changes in these indicators imply the involvement of humors in the process of RIPC-induced ischemic tolerance. The application of ganglion blockers abolished the protective effect of RIPC, suggesting the importance of neural pathway integrity [157].

#### 4.3.2 Preconditioning of mesenchymal stem cells (MSCs)

Mesenchymal stem cells (MSCs, or mesenchymal stromal cells) are an important member of the stem cell family, originally found in bone marrow (BM), but also derived from adipose tissue (AT), placenta, umbilical cord (UC), and dental pulp (DP). MSCs are valued in regenerative therapy for their powerful proliferation, differentiation, immunomodulation, and paracrine functions, as a promising therapeutic approach to promote recovery after ischemic stroke [158]. The neuroprotective effects of MSC transplantation have been frequently explored in numerous preclinical studies in vivo and in vitro. MSCs inhibit inflammatory responses and apoptosis to promote the survival of injured neurons. Furthermore, MSCs can secrete soluble factors and extracellular vesicles (EVs) [159], the former including various trophic factors, growth factors, and cytokines, and the latter including exosomes, macrovesicles and apoptotic vesicles. EVs are loaded with a variety of beneficial proteins, lipids, nucleic acids, etc. These beneficial secretions are capable of promoting angiogenesis, protecting neural tissue, and repairing damaged neurons.

The therapeutic potential of MSCs is diminished by the poor survival rate due to the harsh microenvironment of the lesion. Researchers have been looking for new strategies to improve the therapeutic efficiency of transplanted cells, and preconditioning of MSCs is a promising choice. The role of preconditioning for MSCs has two concerns: one is to promote the survival of MSCs in the I/R or OGD/R environment, and the other is to enhance the therapeutic effect of MSCs or MSC-conditioned medium (MSC-CM), covering the enhancement of beneficial paracrine activity. In fact, researchers have explored various biological, physical, chemical, and pharmacological preconditioning factors, among which hypoxic preconditioning has been the most frequently studied.

In vitro studies showed that hypoxic preconditioning of BMMSCs resulted in higher proliferation rates, lower mortality, and more delayed senescence [160]. Rat cytokine arrays showed significant changes in paracrine secretion, with higher expression of nine proteins (including VEGF, TIMP-1, MCP-1, etc.) and lower expression of leptin and TNFα in conditioned media [161]. Hypoxic preconditioning increased the viability of transplanted UCMSCs under OGD/R conditions and attenuated apoptosis [162]. BMMSC-CM effectively promoted the survival and anti-inflammatory polarization of microglia exposed to OGD/R and mitigated cellular damage. The neuroprotective and anti-inflammatory properties of paracrine factors were further heightened by hypoxic preconditioning [163]. In addition, it was found that VEGF secretion was stimulated by hypoxic preconditioning in aged BMSCs, augmenting their therapeutic effect [164]. In rat MCAO models, hypoxic preconditioning strengthened the effect of BM-MSCs in the treatment of cerebral infarction, further reducing neuronal apoptosis, facilitating cell survival, engendering regeneration, and restoring neurological functions [165,166]. In a mouse focal I/R model, hypoxic preconditioning potentiated the neurorepair efficiency of small EVs from MSCs, which was associated with increased angiogenesis and decreased delayed neurodegeneration [167]. Moreover, the enhanced therapeutic efficacy of MSCs or MSCs-CM by hypoxic preconditioning has been observed in models of TBI and NHIE [[168], [169], [170]].

In addition, preconditioning with other factors has shown positive effects, including but not limited to (1) pharmacological and chemical agents, such as hydrogen sulfide [171], lipopolysaccharide [172], sevoflurane [173], melatonin [174], celastrol [175], and thrombin [176]. (2) Micronutrients, such as zinc [177], and lithium [178]. (3) Tissue extracts, such as cerebral infarct tissue extracts [179], and traumatically injured brain tissue extracts [180]. (4) Conditioned medium such as dental pulp stem cell (DPSC)-CM, and hair follicle stem cell (HFSC)-CM [181]. (5) Cytokines, such as polarizing cytokines, TNF-α, and IFN-γ [182,183].

Preconditioning of MSCs is a novel method whereby therapeutic stem cells are preconditioned in vitro, dramatically reducing the potential risk to brain tissue from the preconditioning itself. The regenerative effects of stem cells facilitate a shift in neuroprotective strategies of preconditioning from reducing cell death to promoting neurological recovery. Preconditioning of MSCs shows great potential for clinical application, but also faces many challenges to be overcome, such as the various risks associated with transplantation, the low survival rate of transplanted MSCs, and the inadequate study of preconditioning. More in-depth studies are still needed to explore standardized protocols for preconditioning, optimal administration pathways, accurate dosing, etc.

#### 4.3.3 Inflammation

The burst of research on inflammatory mechanisms in recent years is partly attributed to the increased exploration of the role of peripheral immunity in preconditioning. A study published in 2016 demonstrated a correlation between RIPC-induced ischemic tolerance and changes in peripheral immunity [25]. RIPC reduced the infiltration of CD3^+^CD8^+^ T cells after stroke and increased the proportion of protective B cells and noninflammatory monocytes in peripheral blood, providing neuroprotection in ischemic areas. This conclusion is supported by another study in which RIPC significantly ameliorated the reduction in T cells (CD4 and CD8) in peripheral blood, inhibited the infiltration of T cells (CD4 and CD8), and enhanced the infiltration of B cells in the brain after I/R in type 2 diabetic mouse model of MCAO [184], suggesting that preconditioning may attenuate inflammatory injury by modulating the two-way communication of the immune system between peripheral blood and brain tissue. Ischemic preconditioning attenuated the elevation of inflammatory cells (e.g., monocytes and neutrophils) together with inflammatory mediators (e.g., miR-329-5p) in the blood of stroke mice and promoted the expression of the M2 marker YM1 in the ischemic hemisphere. Consequently, it elevated the anti-inflammatory/proinflammatory ratio in ischemic areas, reducing infarct volume and brain edema [185]. In a prospective cohort study, patients with TIAs within 7 days prior to stroke had lower blood levels of IL-6, supporting that ischemic preconditioning can reduce the systemic inflammatory response. In addition, repetitive hypoxic preconditioning decreased peripheral leukocyte infiltration into ischemic areas to protect brain tissue from inflammatory damage by upregulating the number of C-X-C motif chemokine ligand 12 positive (CXCL12^+^) microvessels [186].

Excessive inflammatory responses in the central nervous system are key pathological mechanisms mediating cerebral ischemia/reperfusion injury, with many inflammatory cells and mediators involved. It is believed that inhibiting proinflammation and increasing anti-inflammation are important strategies for reducing brain damage. At the cellular level, microglia are the main effector cells during ischemia/reperfusion injury. Sevoflurane and hypoxic preconditioning could promote the polarization of microglia towards an anti-inflammatory phenotype to minimize inflammatory damage and protect neural tissue [56,187]. Although AP39 (a slow-releasing and mitochondrion-targeted hydrogen sulfide delivery molecule) preconditioning did not promote the polarization of microglia to the M2 anti-inflammatory phenotype, it inhibited the polarization of microglia to the M1 proinflammatory phenotype and reduced the expression of IL-1β, IL-6, and TNF α, exerting a significant anti-inflammatory effect [188]. Microglial accumulation and activation are important factors in secondary inflammatory injury after SAH. LPS preconditioning significantly diminished microglia accumulation and activation to decrease secondary neuronal injury and death [189]. Immunoregulation is part of the interaction between astrocytes and neurons. It was shown that IPC exerted anti-inflammatory effects by activating the astrocyte TLR3/TRIF/pIRF3 signalling pathway, increasing IFNb secretion and inhibiting IL-6 secretion [190].

At the molecular level, downstream proinflammatory factors (e.g., IL-1β, IL-6, and TNFα) and anti-inflammatory factors (e.g., IL-10, TGFβ, and type I interferon) are important indicators of the inflammatory response, while TLRs and inflammasomes are important upstream inflammatory mediators in the central nervous system. By altering the TLR cascade signalling pathway after cerebral ischemia, preconditioning was able to promote the expression of anti-inflammatory cytokines and inhibit the expression of proinflammatory cytokines to reduce inflammatory damage and protect neural tissue. Among all TLRs, knockout of TLR4 abolished the neuroprotection induced by ischemic preconditioning, suggesting that TLR4 is essential for ischemic tolerance. Mechanisms involving TLR4 focus on the regulation of the proinflammatory signalling pathway TLR4//MyD88/NF-κB and the anti-inflammatory signalling pathway TLR4/TRIF/type I interferon [191]. Preconditioning with ethanol extracts from *Ilex pubescens* (iPee) induced ischemic tolerance by suppressing the MyD88-dependent pro-inflammatory pathway and activating the TRIF-dependent anti-inflammatory pathway [192]. As an integral part of cerebral I/R injury, the activation of the NOD-like receptor protein 3 (NLRP3) inflammasome leads to pyroptosis, a proinflammatory form of cell death [193]. In a mouse ischemia/reperfusion model, hypoxic preconditioning decreased pyroptosis to prevent inflammatory injury by inhibiting the expression of the NLRP3 inflammasome and the related proteins Caspase-1 and Gasdermin D [194]. This protection has also been observed in exercise preconditioning. Exercise preconditioning inhibited the protein expression of NLRP3, Caspase-1, IL-18, and IL-1β to improve cognitive dysfunction in stroke mice [195]. Moreover, 30-min desflurane preconditioning promoted the survival of human umbilical vein endothelial cells in hypoxia/reperfusion in vitro, partly attributed to the diminution of the inflammatory response via upregulation of NLRP12 expression and inhibition of NF-κB expression [196].

## 5 Limitations

The following limitations of this study must be noted: First, as preconditioning and pretreatment are extremely confusing [5], we have only searched for preconditioning and may have missed some important studies. Second, we only searched the WOSCC database for research articles in English, because CiteSpace was developed based on the WOS database. Other databases only allow for partial analysis; for example, PubMed and CNKI do not support citation analysis. However, WOSCC has a wide coverage, and its data can represent most information. Therefore, we still consider the findings to be reliable.

## 6 Conclusion

In this study, we used CiteSpace software for the first time to visually analyse the literature in the field of preconditioning in cerebral ischemia from the WOSCC database between 1999 and 2022.

The findings revealed that the annual number of publications showed an upwards and then downwards trend, but currently remained high in terms of annual publications. The US was the leading country, and Perez-Pinzon, Miguel contributed the most publications. China was the most active country in recent years, and Capital Medical University published the largest number of articles. KITAGAWA K from Japan was the most cited author. The focuses of the study were summarized in the following three areas: (1) relevant diseases and experimental models, (2) types of preconditioning and stimuli, and (3) mechanisms of ischemic tolerance. Research frontiers included RIPC, preconditioning of MSCs and inflammation. Noninvasive RIPC is safer and ethically compliant for clinical research and application. Preconditioning of MSCs is a new type of preconditioning for therapeutic tools that aims to improve the properties of MSCs. These two types of preconditioning represent a new research trend in which safe, clinically feasible preconditioning is becoming predominant. Further in-depth investigations are needed in the future to explore these promising preconditioning strategies.

Our study provides a visual and scientific overview of research on preconditioning in cerebral ischemia, valuable information and new directions for researchers.

## Funding

This work was supported by the 10.13039/501100007129Natural Science Foundation of Shandong Province (ZR2020MH156).

## Ethics statement

Review and/or approval by an ethics committee was not needed for this study because this is a bibliometric analysis based on other studies.

## Data availability statement

All data are included in the article and its supplementary information files. All data can be generated from files in the supplementary material. Further inquiries can be directed to the corresponding author.

## CRediT authorship contribution statement

**Long Zhang:** Writing – original draft, Visualization, Software, Methodology, Formal analysis, Conceptualization. **Xue Zhou:** Methodology, Formal analysis, Data curation. **Jing Zhao:** Methodology, Formal analysis, Data curation. **Xingchen Wang:** Writing – review & editing, Validation, Supervision, Conceptualization.

## Declaration of competing interest

The authors declare that they have no known competing financial interests or personal relationships that could have appeared to influence the work reported in this paper.
